# Scalable production of large components by industrial robots and machine tools through segmentation

**DOI:** 10.3389/frobt.2022.1021755

**Published:** 2022-12-14

**Authors:** Thorben Schnellhardt, Rico Hemschik, Arno Weiß, Rene Schoesau, Arvid Hellmich, Steffen Ihlenfeldt

**Affiliations:** ^1^ Fraunhofer Institute for Machine Tools and Forming Technology IWU, Dresden, Germany; ^2^ Fraunhofer Institute for Material and Beam Technology IWS, Dresden, Germany

**Keywords:** segmented manufacturing, large component, CNC machining, process planning, mobile machine tool, laser metal deposition

## Abstract

The production of large components currently requires cost-intensive special machine tools with large workspaces. The corresponding process chains are usually sequential and hard to scale. Furthermore, large components are usually manufactured in small batches; consequently, the planning effort has a significant share in the manufacturing costs. This paper presents a novel approach for manufacturing large components by industrial robots and machine tools through segmented manufacturing. This leads to a decoupling of component size and necessary workspace and enables a new type of flexible and scalable manufacturing system. The presented solution is based on the automatic segmentation of the CAD model of the component into segments, which are provided with predefined connection elements. The proposed segmentation strategy divides the part into segments whose structural design is adapted to the capabilities (workspace, axis configuration, etc.) of the field components available on the shopfloor. The capabilities are provided by specific information models containing a self-description. The process planning step of each segment is automated by utilizing the similarity of the segments and the self-description of the corresponding field component. The result is a transformation of a batch size one production into an automated quasi-serial production of the segments. To generate the final component geometry, the individual segments are mounted and joined by robot-guided Direct Energy Deposition. The final surface finish is achieved by post-processing using a mobile machine tool coupled to the component. The entire approach is demonstrated along the process chain for manufacturing a forming tool.

## 1 Introduction

The demand for large and accurately machined parts is rising. Currently, those parts are usually machined on large machine tools. However, large machine tools suffer from some significant shortcomings (Uriarte et al., 2013), i.e. high level of investment, low sustainability due to high energy and material consumption, low productivity due to long machining cycle times and several technical limitations that arise from the large dimension/workspace size (e.g. thermal issues, reduced stiffness). To address these shortcomings various approaches exist in the research field of machine tools. E.g. optimized machine structures to improve the eco-efficiency (Zulaika and Campa, 2009), size-scalable machine tool frames based on polyhedral building blocks that enable reconfigurability (Uhlmann and Peukert, 2019) or mobile machine tool solutions (Neugebauer et al., 2012) that improve the utilization of resources. However, most approaches have not yet found their way into industry or target only a subset of the aforementioned issues. When compared to conventional machine tools, industrial robots have a large workspace that can be expanded further. DeVlieg (2011) and Saund and DeVlieg (2013), for instance, extend a robot with an additional linear axis for the machining of large-scale aluminum aircraft components. Möller et al. (2017) and Susemihl et al. (2017), on the other hand, developed a mobile robot system on an autonomous mobile platform, capable of machining composite (CFRP) aircraft components. An advantage of such mobile robotics solutions is the possibility of scaling and parallelization, which can significantly reduce process times.

Although robots are currently used in the machining of large components, they are significantly less rigid compared to conventional machine tools (static Cartesian stiffness is up to 50 times lower), which reduces machining accuracy, hence they cannot be utilized for all applications (Verl et al., 2019).

To overcome this problem, numerous approaches can be found in the literature, including the design optimization of milling robots (Denkena et al., 2017), various concepts for structural optimization (Tao et al., 2019) or placement optimization (Xue et al., 2022). However, robotic milling in large-scale component manufacturing is currently limited to softer materials such as aluminum, plastic, and composite (Kim et al., 2019). This excludes components with high hardness and accuracy requirements such as forming tools.

To address this problem, we propose a novel and sustainable approach to large component manufacturing that enables robots and regular sized machine tools to manufacture large components (with high hardness and accuracy requirements) within a highly scalable manufacturing system. Thus reducing the need for expensive and resource intensive large machine tools. The approach is based on segmented manufacturing and subsequent joining of the large component. Key to the approach is the segmentation of the component into segments that are tailored to the capabilities of the available shop floor entities, i.e., machine tools and robots equipped with different end effectors and tools.

So far, approaches towards segmented manufacturing are sparse in the literature. Ley et al. (2018) present an approach for hybrid-optimized manufacturing of large components by segmenting the component into subcomponents. However, their focus is primarily on the combination of additive and conventional manufacturing and less on automation and productivity. Further examples of segmented manufacturing can be found in the area of toolmaking. These include mold inserts in forming tools (Cao et al., 2019) or multi-part injection molds (Stoyan and Chen, 2010). However, their focus is primarily on the functionalization of the component rather than the actual manufacturing process.

The main goal of this paper is to present the basic principles of the proposed approach. For this purpose, a general overview of the underlying process chain is given in Section 2.1. Section 2.2 presents the conceptual architecture as an essential enabler for the modularity and scalability of the approach. Section 2.3 describes the segmentation procedure. Joining and finishing of the segments are discussed in Section 2.4 and Section 2.5. Finally, the basic manufacturing approach is demonstrated and validated on a downscaled forming tool.

## 2 Methods

### 2.1 General

**FIGURE 1:**
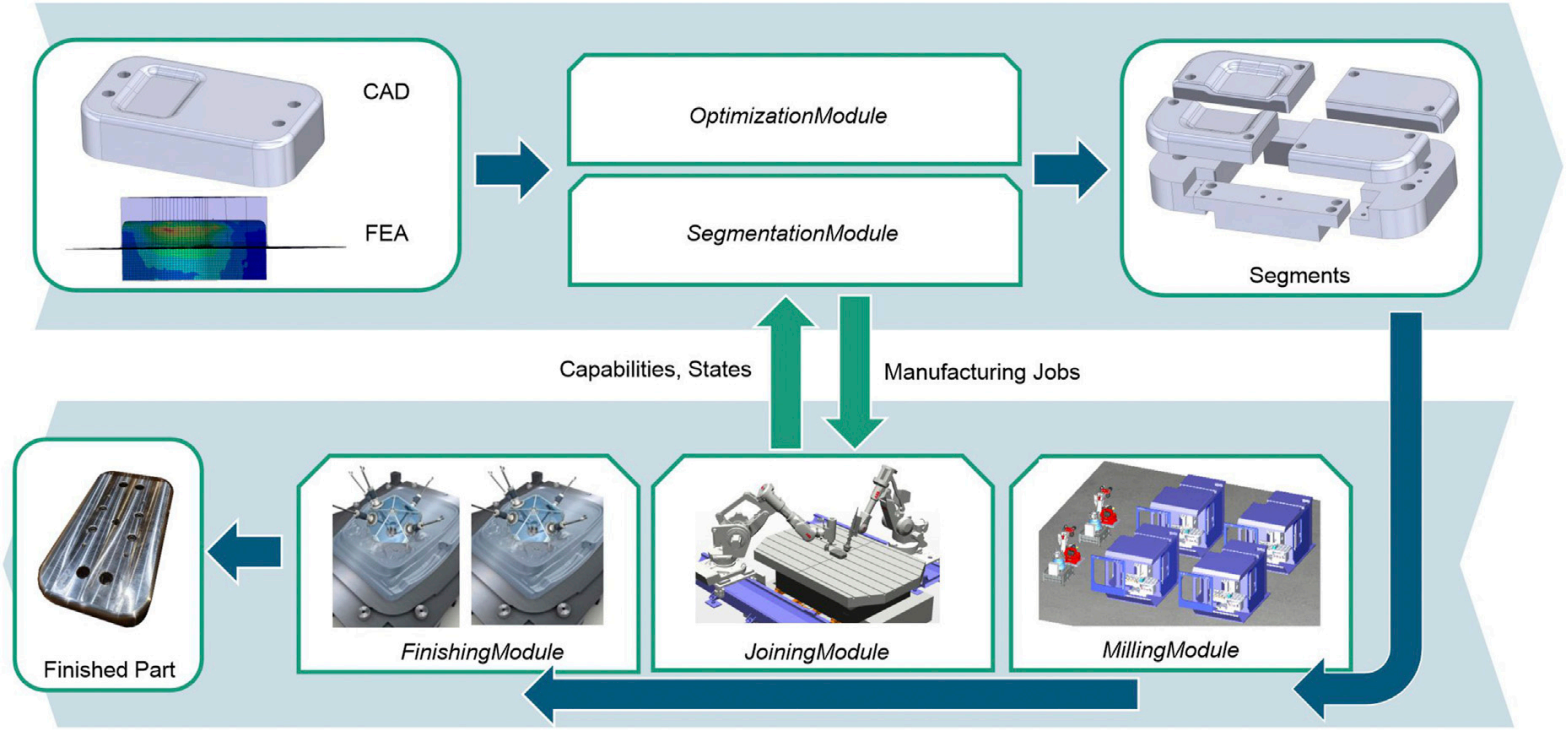
Overview of the approach and the underlying process chain.

This section provides an overview of the proposed approach. Figure 1 depicts the basic process steps of the approach in terms of the involved modules. Modules (see Section 2.2) have a specific task in the manufacturing system and represent the necessary software and hardware entities (assets). Modules are modeled by lightweight information models, which they expose *via* uniform communication interfaces. These information models describe the capabilities, states, and services of the assets. The considered manufacturing system consists of a set of loosely coupled machine tools and robots, equipped with different end effectors and tools.

Starting point of the manufacturing process is the CAD model and a Finite Element Analysis (FEA) of the large component. The *SegmentationModule* applies a predefined, application specific segmentation strategy and splits the CAD model into a combination of smaller, less complex segments. The segments are split in a way, that optimizes a given target quantity, e.g., time, cost or utilization rate. Therefore, the *SegmentationModule* provides an interface to an *OptimizationModule*, which determines a combination of segments that suits the capabilities of the existing assets and maps them accordingly. The CAD models of the individual segments are extended with connection elements (e.g. screw connections) for assembly. The connection elements are part of the segmentation strategy. Afterwards, NC programs are created for the individual segments using a feature-based approach with predefined machining operations. The NC programs are passed to the corresponding *MillingModules* and are manufactured. *MillingModules* represent assets such as conventional CNC machines or milling robot cells.

The manufactured segments are assembled and thermally joined by the *JoiningModule* to bridge the segment gaps induced by segmentation. Thus, a robot-guided Direct Energy Deposition (DPD) process is used at this point. The aim is to minimize component distortion in order to reduce the amount of post-processing required during the final stage of the process. After the joining, the component is measured *in situ* by the robot to determine the finishing effort. The measurement data is finally used for the finishing process, which is performed by mobile machine tools (*FinishingModules*) that are temporarily coupled to the component.

### 2.2 Architecture

Executing the previously described process is primarily a technological task with high requirements to process quality. However, the targets motivating this novel approach (scalability, productivity) can only be met with comprehensive automated planning and execution. A particular challenge arises from the inherent heterogeneity of production assets involved. They differ with regard to their scope (i.e., segmentation, production, joining), with regard to their origin (vendors, PLCs) and with regard to their age. All these factors influence the interfaces available to upper-level process coordination in the *OptimizationModule*. An architecture suitable for the task must handle this complexity, thus automate the material and information flow between production assets.

To meet these requirements, we followed the integration guide of the SWAP-IT architecture (Lünsch et al., 2022). The SWAP-IT architecture is composed of autonomous *Modules*, each of which specifies its characteristic *Services* to peers. A *Service* signifies the *Module’s* potential to trigger an executable process, similar to a *Skill* in literature (Köcher et al., 2020). The *Module Services* are implemented by its member *Agents.* Each *Agent* encapsulates an asset (logical or physical) and adapts the proprietary communication interface to a harmonized representation in the network. All *Agents* register with a central entity that provides transparency on available *Agents* and their *Services* in the network. This highly decentralized architecture enables flexible routing and reallocation of resources according to the requirements of the production order and current availability. The communication backbone for interaction between and within modules is OPC UA as it supports the reuse of common semantic information models that are tightly coupled to communication mechanisms for machines and computers alike. These information models are used to represent the *Module*’s specific *Capabilities* and *States*.

As all steps mentioned in Section 2.1 must be represented on the *Module*-level, *Modules* for segmentation, milling, joining and finishing were designed and implemented on *Agents*. The following chapters provide an in-depth description of their functionality.

### 2.3 Segmentation

The proposed approach increases the number of parts to be manufactured compared to conventional large component production. Accordingly, the design and planning effort also increases, which in turn has a negative impact on production costs and duration and thus eventually also has a negative effect on overall productivity. Moreover, manual segmentation is prone to errors, as the complexity and dependencies within the assembly scale with the number of segments. Therefore, it is crucial for the applicability of the approach to automate the segmentation.

**FIGURE 2:**
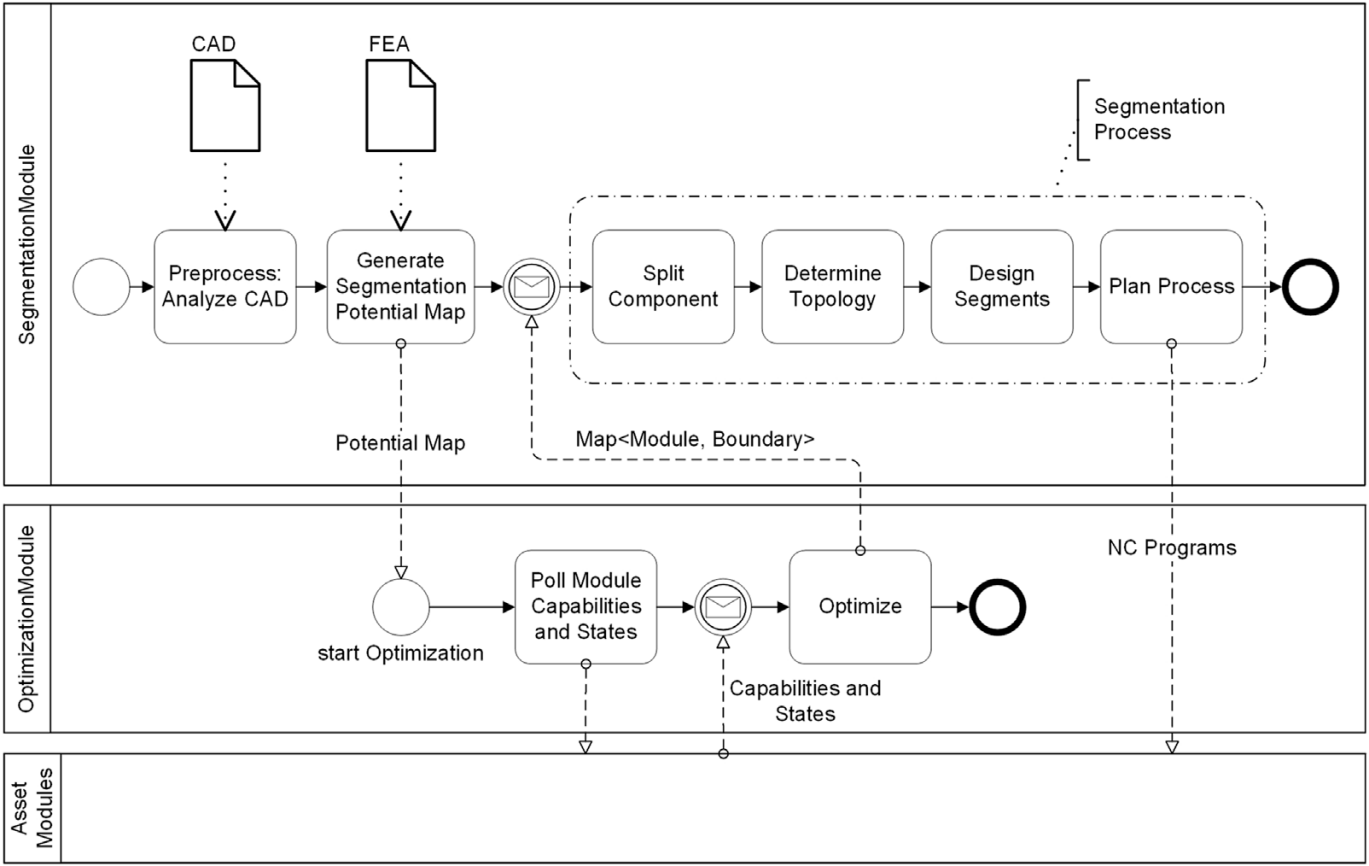
Workflow of the segmentation procedure.

In our case, the *SegmentationModule* handles this task, i.e., the automatic execution of the CAD-CAM chain along the segmentation from the CAD model of the large component, through the design of the individual segments, to the process planning and the NC programs. Thereby, the characteristic properties of the segmentation (similarity of the segments, lower complexity compared to the overall component) are utilized. In addition, the module provides an interface to the *OptimizationModule*, which determines suitable segment combinations. Figure 2 summarizes the process steps involved in the segmentation procedure, which are described in detail in the following.

#### 2.3.1 Preprocess – analyze CAD

In the beginning, an initial part analysis is performed to derive the geometric constraints for the segmentation. For this purpose, an automatic design feature analysis of the CAD model is carried out. The goal of this analysis is to identify those areas of the part that must not be segmented, e.g., connection holes. In the case of a native CAD format, the vendor specific design features can usually be utilized for this task, e.g. we use the design features that SolidWorks provides *via* their API. Alternatively, a surface analysis of the B-Rep model must be conducted using one of the various feature recognition methods (e.g. (Han et al., 2000), (Zhang et al., 2017), (Zhang et al., 2018)). In addition, the part is searched for areas that require 5-axis machining. For this purpose, the surfaces of the CAD model are grouped into standard 2.5D feature surfaces and freeform surfaces according to their B-Rep model.

#### 2.3.2 Generate segmentation potential map

For the optimization phase, the constraints of the segmentation are merged and brought into a uniform format in form of a segmentation potential map. Therefore, a mapping is used which assigns numerical values to the regions of the large component, which describe the segmentation potential of the respective region. To generate these values, the absolute values of the FEA stress data are utilized. The FEA denotes the expected stress during the usage of the final product. To integrate the geometric constraints (from the preprocess stage), the positional data of the geometric constraints are amplified with high values and overlaid with the absolute stress values. This results in a mapping where low absolute values describe a high segmentation potential and high values describe a poor segmentation potential.

#### 2.3.3 Optimization

Input of the *OptimizationModule* is the potential map generated in the previous step. Furthermore, the *Module* uses the *Capabilities* and *States* (workspace, machine costs, axis configuration, availability, etc.) of the *MillingModules.* The goal of the optimizer is to segment the part and map the segments to the available assets in such a way that an optimal production time, cost or utilization rate is achieved. At the same time, the constraints, encoded within the potential map, have to be satisfied. I.e. the boundaries of the segments should only intersect low valued regions of the potential map. The result of the *OptimizationModule* is a segmentation recommendation in form of an assignment of segment boundaries (Axis-Aligned Bounding Boxes) and *MillingModules.*

#### 2.3.4 Split component

In this step, the actual CAD model is finally split into the individual raw segment pieces according to the segmentation recommendation of the *OptimizationModule.* Furthermore, the classification of the segments into specific types is performed in this step, if required by the applied segmentation strategy.

#### 2.3.5 Determine topology

Next, segment topology is determined, i.e., the spatial relationship of the individual segment parts and their geometric entities (faces and edges) to each other. This is necessary for the extension of the segments with the connection elements for the assembly. Furthermore, the topology information is used to apply the tolerances to the segments. Based on the assumption that the intersections between the segments are planar surfaces, a collision check using Oriented Bounding Boxes in 3D/2D (body to body and face to face) is applied for the topology determination.

#### 2.3.6 Design segments

According to the selected segmentation strategy and the face/body relationships (topology), the connection features and tolerances are constructed automatically onto the segments. To simplify CAM planning, only subtractive design features are used during this stage.

#### 2.3.7 Plan process

Based on the data collected during the previous process steps (feature recognition, topology, tolerances) feature-based process planning is performed. Therefore, a matching between predefined machining operations, tools and machining features (based on ISO 14649-10, 2004) is carried out. The basis for this is the similarity of the segments and their reduced complexity compared to the overall component. Segments consist of the planar cut surfaces caused by segmentation, a fraction of the surface of the original component and a number of predefined connection elements. This simplifies the accessibility and clamping of the segments significantly and thus eases the automation potential of the processes. For the generation of the machine-specific NC code, the *MillingModule’s* information models and the corresponding postprocessor are utilized. Finally, the execution of the *MillingModule’s Services* are triggered on the field device using the generated NC-Programs.

### 2.4 Joining

After the individual segments have been manufactured, they are joined by using industrial robots. Here, a DED process is used that applies a powdery filler material to the component’s surfaces with the help of a laser and thus fuses the segments metallurgically. For this purpose, a double robot system is used with two processing heads on which OTS-2 laser optics are mounted. Two COAX Powerline powder nozzles are utilized to inject the powder material into the process zone, each of which are integrated into a COAXshield system (Kolsch et al., 2020) for better shielding of the process. This makes it possible to carry out the welding process within a local shielding gas without having to flood the entire installation space of the cell, which is crucial when processing large components.

The robot cell used in this research offers a usable workspace of 3 m length, 3 m width and 1 m height. Both robot systems are mounted onto linear axes and are able to operate synchronously. In addition, the cell offers a rotary table. The robot’s traversing strategy for the welding process must be automated for the most part to ensure productivity. As the positions of the weld seams are already known from the preceding segment design process of the *SegmentationModule*, the necessary welding trajectories of the robot can be derived directly.

For the alignment of the segments onto the machine table, it is necessary to induce a workflow that avoids clamping errors, since they have a major influence on the final geometry and accuracy of the component. Therefore, two approaches were established. On the one hand, when subdividing the large component, it is important to ensure that the segments produced can be mounted and clamped easily and form-fittingly. On the other hand, it is important to pre-measure the entire geometry of the clamped large component in order to enable precise alignment of the coordinate system. For both measures, it is advisable to provide mounting holes whose arrangement corresponds to the standardized clamping grooves on machine tables. With the aid of these holes, the segments can be positioned quickly and precisely, and a subsequent 3D scan or measurement with a probing device, allows the workpiece coordinate system to be set up precisely.

After the joining process, a 3D measurement of the large component is conducted, in order to measure both the distortion and the geometries of the applied weld seams. The measurement is carried out automatically by the manufacturing robot. For this purpose, a MICRO-EPSILON scanControl 2900–100/BL line scanner is mounted onto the robot. The robot automatically scans the entire component surface with the help of a measuring routine and prepares the data as a STL file. Since the applied line scanner only has a relatively narrow measuring range of approx. 8 cm, the component is scanned several times with overlapping strips. The individual scans are automatically combined by using feature recognition, in order to map the entire surface of the component. For the actual measurement process, it is important to move as few robot axes as possible in order to avoid error entry due to tolerances. Therefore, only the linear axis on which the robot arm is mounted on or the rotary table that holds the component is moved for the measurement.

After a nominal-actual comparison with the existing CAD data of the component, it is possible to detect any distortion and the weld seam geometry for the entire component. Therefore, it is possible to reduce the subsequent finish machining to areas that deviate from the nominal geometry and thus increase the efficiency of the entire production process.

### 2.5 Finishing

The finish machining of the areas deviating from the nominal geometry is carried out with a small mobile machine tool with parallel kinematic design (Georgi et al., 2018) which is temporarily coupled to the workpiece. The main advantage of this mobile machine tool is the possibility of flexible and highly dynamic local machining of large components. Due to the direct positioning of the small machine on the workpiece, the dependency of machine size to workpiece dimension is resolved and downsizing of the production equipment is possible, which in turn increases the transportability and manageability of the production systems as well as the efficiency of the overall system. Due to the necessity that all movement axes have to be on the tool side, the parallel kinematic machine concept proves to be advantageous due to the low moving masses and enables movement with five degrees of freedom.

**FIGURE 3:**
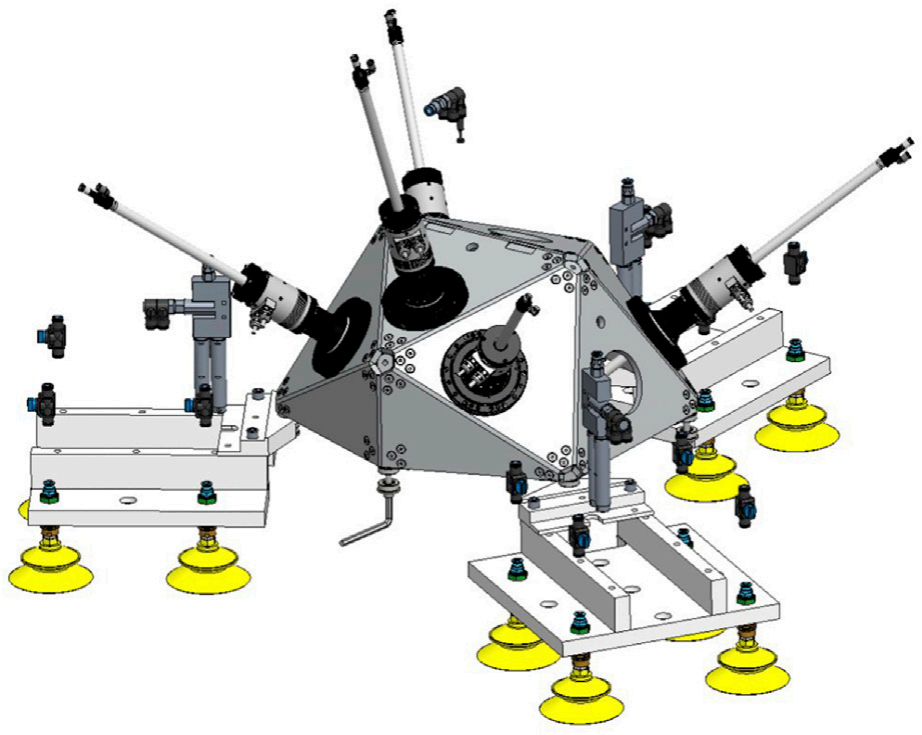
Mobile machine tool with parallel kinematic design equipped with a pneumatic coupling solution.

Solutions for temporarily coupling the mobile machine to the large component are subject to certain design constraints. For example, magnetic clamping systems reach their limits due to their principle when processing aluminium or CFRP components. Thus, a pneumatic coupling solution (Figure 3) consisting of twelve bellows suction cups with upstream rubber joints was developed to compensate for unevenness and displacement due to concave or convex workpiece contours.

The workflow for finishing the areas deviating from the nominal geometry is based on the data from the scan at the end of the joining process. The miniaturized machine tool is positioned over the areas in question. The coordinate systems of the machine and the workpiece are precisely aligned with each other, and the deviation areas (e.g. weld seams, segment deviations) are post-processed and finished.

## 3 Results

**FIGURE 4:**
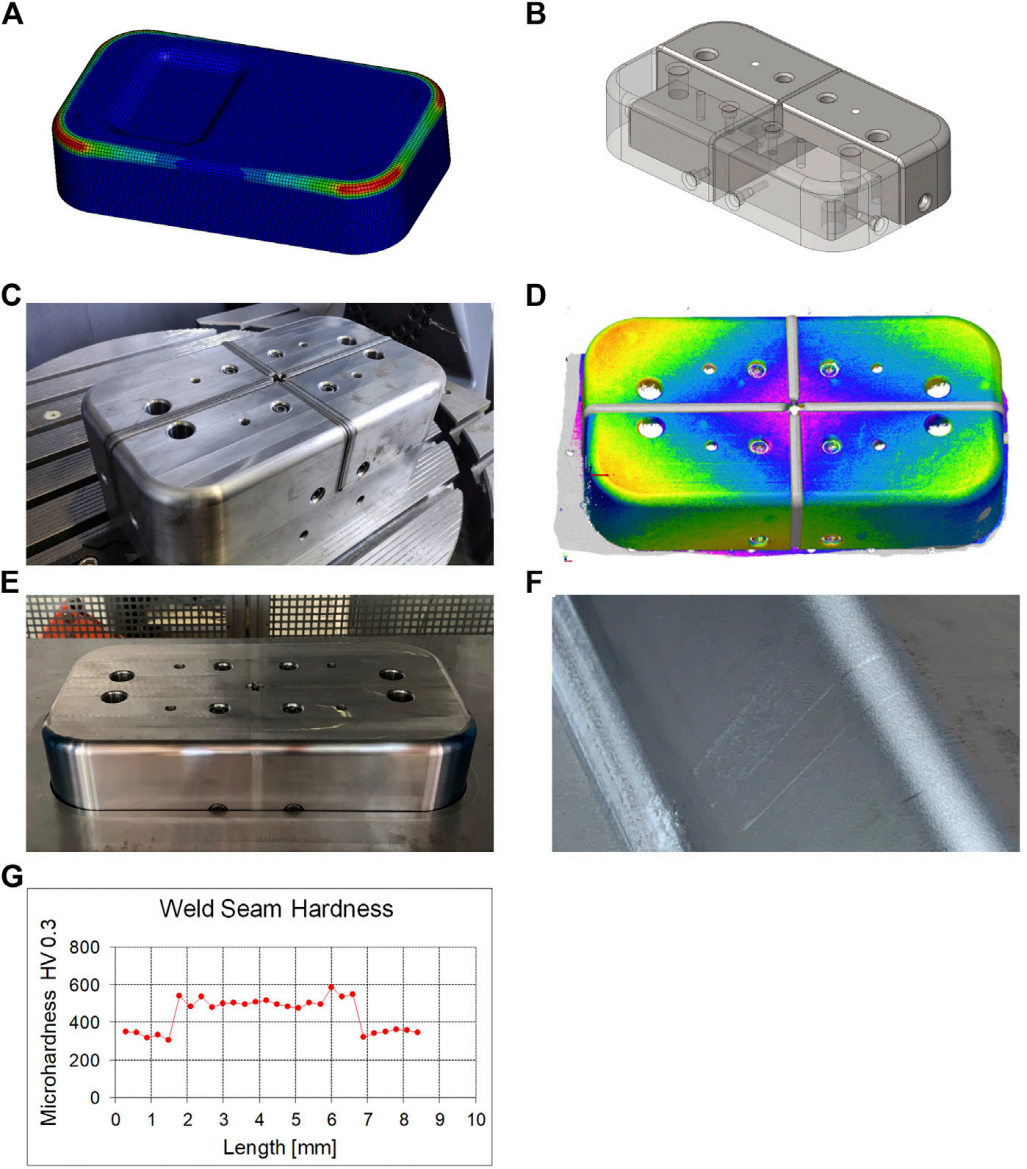
Segmented manufacturing of a deep drawing stamp: **(A)** FEA of the deep drawing process, **(B)** segmented part that consist out of (inner) core- and (outer) shell-segments assembled *via* screw connections, **(C)** assembled and welded stamp, **(D)** distortion of the stamp after the welding process, **(E)** finished stamp, **(F)** marks from the weld seams on the sheet after deep drawing, **(G)** hardness measurement of the seam and the heat affected zone.

The validation of the proposed approach has been implemented and applied on the manufacturing process of a downscaled forming tool. Thereby, the die punch of a rectangular deep drawing tool (347.36 mm × 197.36 mm × 76 mm) served as the test object. The goal of the validation was to investigate the basic applicability of the approach for highly loaded (large) components. Figure 4 shows the individual steps of the validation.

To determine the process-related stresses, a forming simulation was performed using LS-Dyna (Figure 4A). In order to calculate the stress distribution of the die punch, the simulation was carried out with an elastically modeled die. Therefore the elastic tool method of (Haufe et al., 2008) was applied.

The selected segmentation strategy involves the division of the component into shell and core segments (Figure 4B). Core segments are screwed together to form a framework that provides the basic component stability. Shell segments contain the functional surfaces of the component. These segments are attached to the framework of core segments using screw connections. To control the tolerance chain, the shell segments are provided with a clearance fit. The minimal gap between the segments is later closed through the joining process. The segmentation strategy was implemented in the CAD/CAM part of the *SegmentationModule* by using the SolidWorks API.

Toolox 33 prehardened steel was selected as the workpiece material for the punch, in order to avoid a subsequent hardening process in the assembled and joined condition. In a preliminary material characterization study, 1.4404/316L (CrNiMo) and 1.4057/431 (CrNiFe) were analyzed to find a suitable filler material for the DPD process. Due to lower hardening around the heat input zone and the weld seam, 1.4404/316L (CrNiMo) was chosen as filler material for joining the punch. The chosen weld depth was 5.5 mm. The welding was carried out with the robot cell described in Section 3.3. The result is depicted in Figure 4C.

To determine the component distortion, the component was measured optically by a laser line scanner (see Section 2.3) before and after the welding process. This resulted in a distortion of +0.3 mm at the outer edges of the component and a distortion of -0.3 mm in the center of the component (Figure 4D). In addition, a hardness test was carried out in the areas of the heat input zone and the seam. This resulted in a hardening of approximately 200 HV as shown in Figure 4G.

Based on the measurement data, post-processing was carried out to generate the final contour and surface quality (Figure 4E). Therefore, the mobile machine tool was manually aligned on the workpiece in five different clamping setups in order to machine the entire surface. To investigate the functional capability and performance of the segmented die, it was tested in a deep-drawing test series. The sheet material used was DC01 with a thickness of 1 mm. The segmented die withstood the process load and delivered comparable production results to a monoblock die of the same type. As can be seen in Figure 4F, however, the forming process applied light marks to those areas that were in contact with the post-processed weld seams. We attributed these marks to the hardening of the seams that results from the welding process.

## 4 Discussion

A new approach to manufacturing large components by regular-sized robots and machine tools was presented. The approach is based on dividing the large component into segments tailored to the capabilities of the available assets. The segments are joined and post-processed with a mobile machine tool to obtain the final contour. The approach implements a scalable architecture that enables massive parallelization in large-scale component manufacturing.

The associated process chain was validated on a minimal example (forming tool) yielding comparable results to a conventional part. We conclude that the approach is in principle suitable and can increase sustainability and productivity in large component manufacturing due to its scalability. In addition, the approach opens up the possibility for distributed manufacturing at different locations. Furthermore, it mitigates availability problems and reduces the need for cost-intensive special purpose large machine tools in exchange for flexible industrial robots.

A downside is the increased number of necessary manufacturing processes in comparison to conventional process chains. This induces additional complexity. Another shortcoming is the need for an application-specific segmentation strategy, which must be investigated and developed. A general transferability is usually not given and must be examined in each individual case. In the forming tool domain, the approach is currently only suitable for components with limited surface finish requirements, due to the emerging marks on the sheet.

Future work will focus on the implementation of the entire method for a more complex use case where the advantages of the method regarding productivity and scalability can be utilized. In doing so, the potential of the method in its entirety will be investigated. Furthermore, technological fine-tuning is necessary, mainly in the area of the joining and finishing process. Tasks here are the homogenization of the hardening process, the reduction of distortion to reduce the necessary finishing effort and the (semi-)automation of the positioning and alignment of the mobile machine tool.

## Data Availability

The raw data supporting the conclusions of this article will be made available by the authors, without undue reservation.
